# Minimal theory of 3D vision: new approach to visual scale and visual shape

**DOI:** 10.1098/rstb.2021.0455

**Published:** 2023-01-30

**Authors:** Paul Linton

**Affiliations:** ^1^ Presidential Scholars in Society and Neuroscience, Center for Science and Society, Columbia University, New York, NY 10027, USA; ^2^ Italian Academy for Advanced Studies in America, Columbia University, New York, NY 10027, USA; ^3^ Visual Inference Lab, Zuckerman Mind Brain Behavior Institute, Columbia University, New York, NY 10027, USA

**Keywords:** 3D vision, stereo vision, visual scale, visual shape, visual direction, visual constancies

## Abstract

Since Kepler and Descartes in the early-1600s, vision science has been committed to a triangulation model of stereo vision. But in the early-1800s, we realized that disparities are responsible for stereo vision. And we have spent the past 200 years trying to shoe-horn disparities back into the triangulation account. The first part of this article argues that this is a mistake, and that stereo vision is a solution to a different problem: the eradication of rivalry between the two retinal images, rather than the triangulation of objects in space. This leads to a ‘minimal theory of 3D vision’, where 3D vision is no longer tied to estimating the scale, shape, and direction of objects in the world. The second part of this article then asks whether the other aspects of 3D vision, which go beyond stereo vision, really operate at the same level of visual experience as stereo vision? I argue they do not. Whilst we want a theory of real-world 3D vision, the literature risks giving us a theory of picture perception instead. And I argue for a two-stage theory, where our purely internal ‘minimal’ 3D percept (from stereo vision) is linked to the world through cognition.

This article is part of a discussion meeting issue ‘New approaches to 3D vision’.

## 1. Stereo vision

### (a) Failures of triangulation

**Figure 1.**
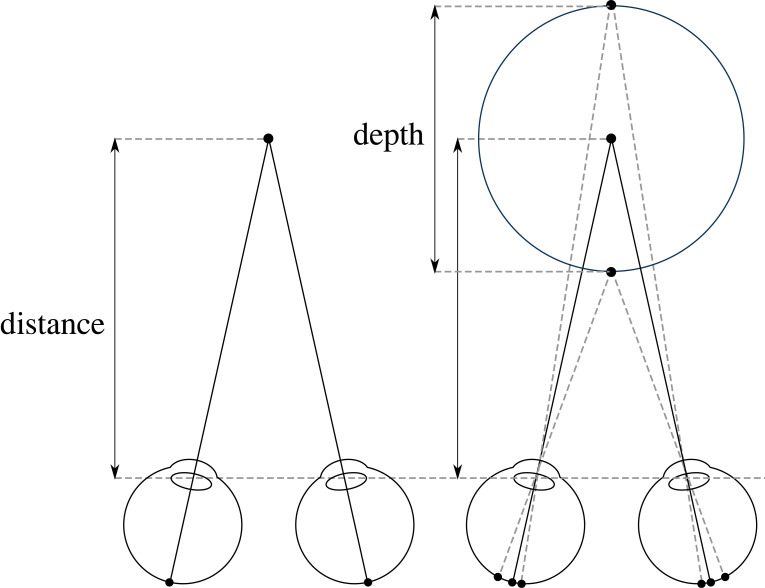
Triangulation model of stereo distance and depth perception. Left: Kepler and Descartes' model of distance perception using vergence (the rotation of the eyes). Right: Wheatstone incorporating disparity (dashed lines) into the triangulation model.

Since Kepler [1] and Descartes [2] in the early-1600s, we've had a ‘triangulation’ model of distance from the two eyes (figure 1, left). So, when Wheatstone [3,4] found out in the early-1800s that stereo vision relied on disparities, this was immediately incorporated into the triangulation account (dashed lines in figure 1, right). As Marr [5, p.110] explains: ‘In stereopsis … the first step is the matching process … the second is the trigonometry that recovers distance and surface orientation from disparity. The first step is the difficult one; the second is easy.’

**Figure 2.**
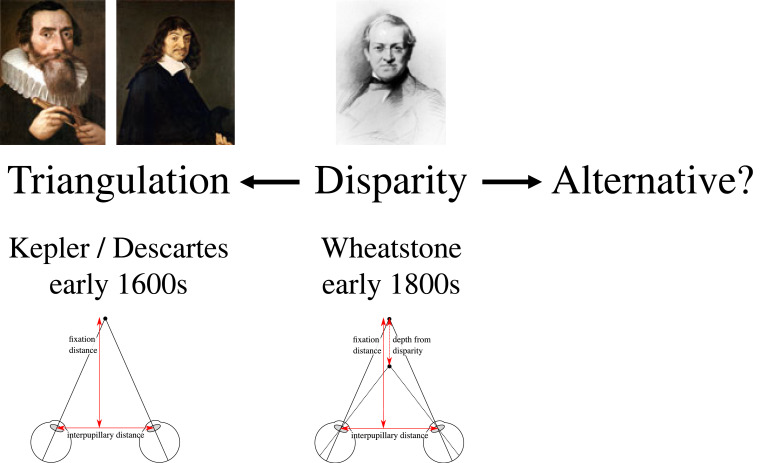
The question is whether we continue to shoe-horn disparities into the triangulation account, or explore my alternative, the rivalry eradication account.

The argument of this section is that it was a historical mistake that we had Kepler and Descartes' triangulation theory of vision (in the early-1600s) for 200 years before we understood that stereo vision relies on retinal disparities (in the early-1800s). And it feels like we've spent the past 200 years trying to shoe-horn disparities back into the triangulation account when they really don't seem to fit (figure 2).

### (b) Problems with triangulation: distance perception

#### (i) Vergence

In order to estimate distance in figure 1 (left), you need to know the rotation of the eyes (vergence). Until recently it was thought that vergence provides ‘critically important information about object distance’ [6] and ‘dominates [reaching] with minimal contribution from pictorial cues’ [7] (see [8–10]). However, in [11], I tested the ability of observers to judge distances using vergence and found they were effectively guessing. In a follow-up study, I also showed that vergence has no effect on perceived size [12]. So what we need, instead, is a purely optical account of stereo vision that only relies on the retinal image.

#### (ii) Vertical disparities

An alternative approach is to take three or more points in the scene and triangulate their relationship to the eyes (‘vertical disparities’ [13–15]). In practice, this seems to be a poor approach as well. First, the only evidence of vertical disparities affecting distance judgements is in the very specific context of a fronto-parallel surface taking up 60–70° of the visual field, and even then vertical disparities only weakly bias distance judgements [16–19]. Since we rarely encounter surfaces that take up that much of the visual field this can't be how we judge distances. Second, artificially increasing the distance between the two eyes (telestereoscopic viewing) radically alters the apparent scale of the scene. But it shouldn't on this account, since it doesn't affect the distance being triangulated. Third, on this account, one or two dots should provide no distance information, but the addition of a third dot should suddenly transform our perception of distance. But there's no evidence that it does.

### (c) Problems with triangulation: depth perception

#### (i) Impossible horizontal disparities

**Figure 3.**
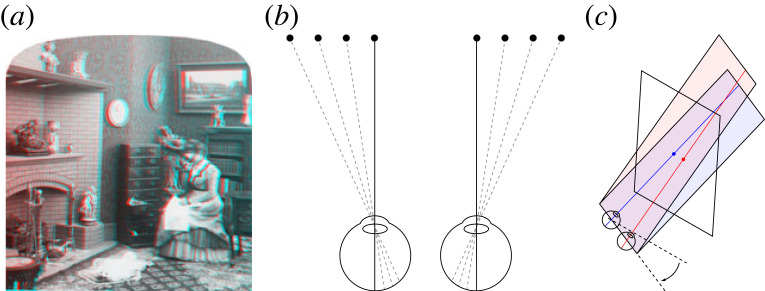
(*a*) Victorian stereogram with uncrossed disparities. (*b*) Resulting impossible horizontal disparities. (*c*) Impossible vertical disparities from head rotation (adapted from [20]). (Online version in colour.)

Stereo photos such as figure 3*a* were wildly popular in the 1800s. But, because the principles of stereoscopy were not well understood, the photographers ended up producing impossible retinal disparities. As we see from figure 3*b*, the projections don't intersect in space, so there's no solution so far as triangulation is concerned. But what's interesting is that we have no problem seeing these stereo photos in depth.

In some ways, this is to be expected. On a triangulation account the distance and direction of points are inextricably linked (figure 1). So, if vision is unable to triangulate distance (from vergence) [11], it's little surprise that it's unable to triangulate direction as well.

#### (ii) Impossible vertical disparities

As we see in figure 3*c*, if the observer tilts their head whilst looking at a stereo display, they will induce impossible vertical disparities. This is where the lines of sight don't intersect because one eye (the right eye in figure 3*c*) is lower, and so is looking upwards. Again, what is interesting is that this doesn't cause any difficulty in seeing stereo depth. And none of the responses in the literature—from intentionally ignoring impossible vertical disparities [21], to averaging out them out [22], to trying to come up with a principled response [20]—make sense on a triangulation account. In particular, [20] are unable to observe the curvature in the stimulus that their explanation predicts.

#### (iii) Vision doesn't delete

**Figure 4.**
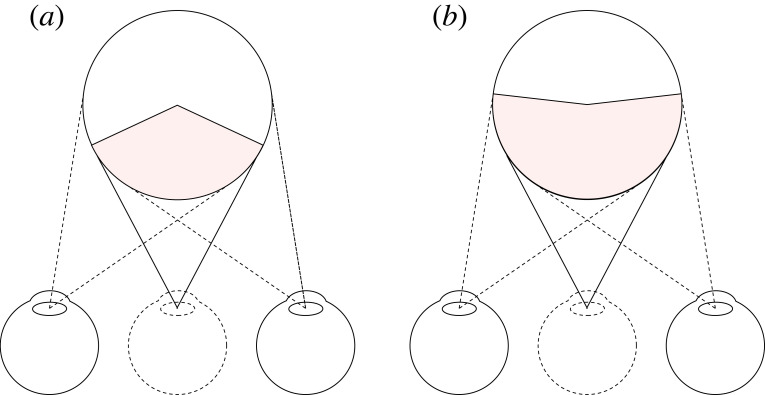
(*a*) Stereo vision on a triangulation account. (*b*) What stereo vision actually appears to do, which is to attribute both retinal images to the cyclopean viewpoint. (Online version in colour.)

According to triangulation, stereo vision is the visual system's version of novel view synthesis [23]. It takes the view from the left eye and the view from the right eye and it synthesizes a new viewpoint midway between the eyes, known as the ‘cyclopean viewpoint’ [24] (dotted eye, figure 4*a*). However, what the visual system actually appears to do, is to take the view from the left eye and the view from the right eye and attribute them both to the ‘cyclopean viewpoint’ (figure 4*b*). Put simply, the visual system doesn't delete.

**Figure 5.**
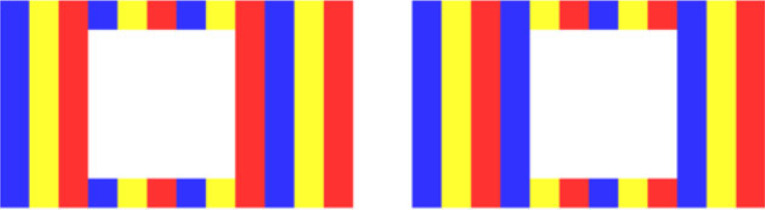
Cross-fuse to see that the visual system attempts to keep the red bar on the right of the white square and the blue bar on the left of the white square. (Online version in colour.)

You can test this by looking at the world and opening and closing one eye. You don't lose any of the retinal image from the other eye. This point is true for all stereo vision, but is immediately apparent in da Vinci stereopsis [25]. For those who can ‘cross-fuse’, in figure 5 you can very clearly see that the visual system attempts to keep the red bar on the right of the white square and the blue bar on left of the white square.

### (d) Stereo depth perception = rivalry eradication

The fact that stereo vision doesn't delete makes no sense on a triangulation account. But perhaps it hints at what stereo vision is doing instead. So far, I've emphasized that the visual system doesn't know the rotation of the eyes, so it is unable to triangulate the distance or direction of points in the world. ‘Not deleting’ goes further and suggests that the visual system doesn't even know that the eyes are in different locations in space. Specifically, not deleting would make a lot of sense if the visual system thought that the eyes were receiving two views from the same location in space.

Indeed, I want to push this thought further and say the visual system isn't thinking about vision in terms of locations in space, or trying to construct models of the world like figure 1. Instead, it's just trying to solve a very different problem: 2D rivalry eradication. The classic example of 2D rivalry is a face in one eye vs. a house in the other [26]. In this example, one image wins over the other. But this is a highly unstable and unsatisfactory solution. By contrast, if the two retinal images are much more similar, does the visual system have an alternative?

**Figure 6.**
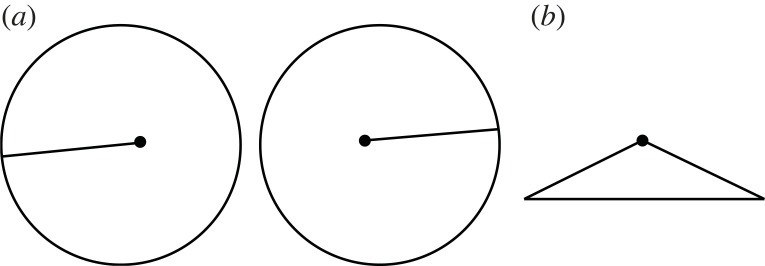
(*a*) It's impossible to find a 2D solution where these two metal rings overlap so that the circles overlap and the points overlap. The answer is to find a 3D solution. (*b*) Side-on view of the 3D solution, bending the arms upwards so the points meet in the middle.

Forget 3D vision for a moment. Imagine I give you two flat metal rings (figure 6*a*), and I ask you to arrange them so that the circles overlap and the points in the centre overlap. Viewed as a 2D problem, this is impossible. If the circles overlap then the points won't overlap, and if the points overlap then the circles won't overlap. But the answer is to realize that there's a 3D solution to this 2D problem. By bending the arms of the metal rings upwards, the points can meet in the middle, with the circles overlapping. Figure 6*b* provides a side-on view of this solution.

And I would argue that stereo vision is doing something similar. On my account, vision is faced with two inconsistent 2D retinal images. Letting them rival one another is the worst-case scenario. So the visual system tries to eradicate their rivalry by finding a 3D solution to this 2D problem, just like figure 6. And this is what you experience as perceived depth off the fixation plane, as points being nearer or further away than fixation^1^.

So, rather than stereo vision being a problem to be solved (how to estimate the 3D properties of the world from the two eyes), stereo vision is the solution to a very different—and purely internal—problem (eradication of rivalry between the two retinal images [27]). The visual system is trying to fit the two retinal images together like a 3D jigsaw to see what emerges.

How is this different from the traditional triangulation account? The key is that this rivalry is unique to my account, because it's a problem caused by the visual system treating the two retinal images as two views from the same location, and therefore vision is faced with a seemingly impossible dilemma of reconciling them. By contrast, on traditional accounts, there is no rivalry to resolve because you can construct a coherent model of the world like figure 1 (although rivalry can inform the initial matching process in the two eyes [28]).

More accurately, on my account, there's no such thing as the location of the eyes, or a model of the world, so far as the visual system is concerned. All vision has is two rivalling 2D images that overlap with one another, and the visual system tries to integrate them (as best it can) into a single coherent percept. It's important to understand that 3D vision solves this problem like a jigsaw; forcing the two pieces together to see what emerges. It has nothing to do with estimating the 3D properties of the world.

### (e) Testing the rivalry account

How could we differentiate these two accounts experimentally?

First, I would argue that the problems I've already raised against the triangulation account: (i) the failure of stereo distance estimation, (ii) impossible horizontal disparities, (iii) impossible vertical disparities, and especially (iv) the fact that stereo vision doesn't delete, are all strong challenges to the triangulation account.

Second, disparities in the real world fall-off as a function of the viewing distance squared. Whether the visual system internalizes this fact is a key differentiator between the ‘triangulation’ and ‘rivalry eradication’ accounts.

**Figure 7.**
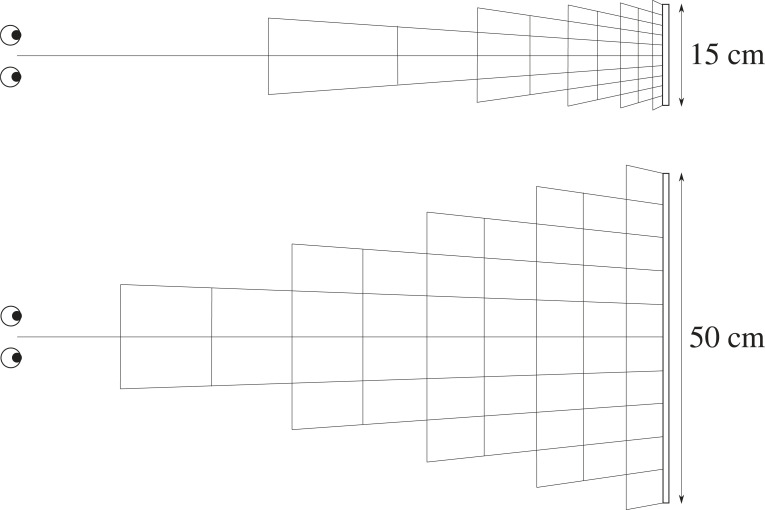
On a triangulation account, changing the size of a stereogram distorts its perceived geometry. Adapted from [22], based on viewing distance 1 m, and stereogram 15 cm vs. 50 cm.

One way to test this is to see if changing the size of a stereogram (while keeping its distance fixed) affects the perceived geometry of the stereogram. On a triangulation account, simply changing the size of a stereogram leads to a distorted percept (figure 7).

By contrast, on a rivalry eradication account, because they have the same disparity, the perceived geometry should remain the same no matter what the size of the stereogram. For instance, if figure 6*b* is the solution to figure 6*a*, then the geometry of the solution should remain the same if we change the size of figure 6*a*.

**Figure 8.**
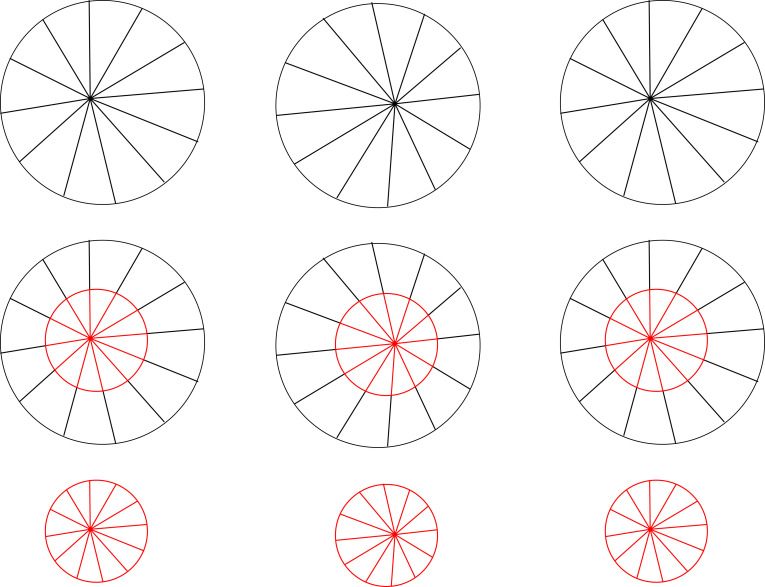
For ‘cross fusion’. A half-size version of a stereogram (in red) looks undistorted against a full-size stereogram (in black). (Online version in colour.)

This is a hypothesis. But how would we go about testing it? Too often the vision literature relies on observers matching shapes, or making judgements about shapes, both of which are taxing cognitive judgements. Instead, a better approach is to embed a miniature stereogram within a larger stereogram and see if it looks distorted. Much will depend on the viewing conditions, but we can at least consider some illustrative examples. For instance, in figure 8, a half-size stereogram looks undistorted against a full-size stereogram, suggesting that changing the size of the stereogram doesn't necessarily distort its perceived geometry.

**Figure 9.**
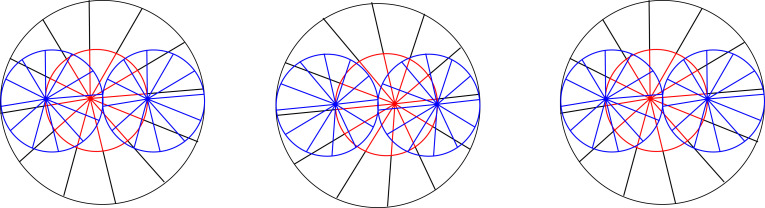
For ‘cross fusion’. The midpoint of half-size stereograms stacked on top of one another (red and blue) appears to coincide with the midpoint of a full-size stereogram (in black). (Online version in colour.)

Another test of this hypothesis is whether the midway point between two half-size stereograms stacked on top of each other is the same as the midway point of a full-size stereogram. This would suggest that depth scales linearly with disparity, so that doubling the disparity doubles the perceived depth. By contrast, because they have the same disparity, on a triangulation account, you might expect the top (nearer) of the stacked half-size stereograms to look flatter than the bottom (further) half-size stereogram. But this doesn't appear to be the case (figure 9), even if you move your face extremely close to the display to maximize the difference.

### (f) Stereo vision ≠ shape processing

It's important to understand that on a rivalry eradication account, stereo vision isn't processing 3D shape. So I talk about ‘depth’ from stereo vision, rather than ‘shape’ from stereo vision.

**Figure 10.**
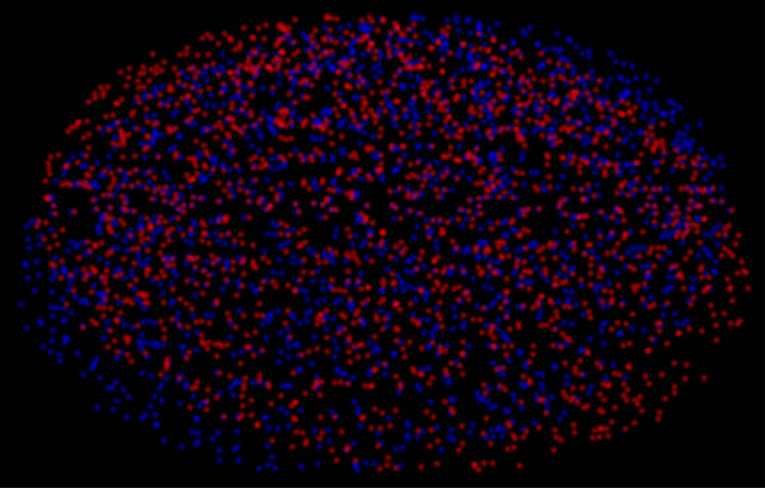
Stereogram from [29]. (Online version in colour.)

Take the stereogram in figure 10. On my account, all that stereo vision is doing is eradicating the rivalry of each individual point by re-locating it in depth. The interpretation of it as a single ‘surface’ is a further, purely cognitive, step [27].

Nor is stereo vision trying to build a 3D model of the world by integrating over time and/or across eye movements or motion. All vision aims to do is resolve the specific rivalry that it is faced with at that specific moment. The clearest illustration of this is the distortion that occurs when you move your head from side to side whilst looking at a stereogram. Try it with figure 10. It feels like the stereogram is following your head movements. This effect is well explained by the fact that the disparity that is hardcoded into the stereogram dictates that the peak of the 3D percept is midway between your eyes (because your right eye sees the peak to its left, and your left eye sees the peak to its right). So, as your head moves, so does the peak [20,22]. There is no attempt to construct a coherent percept across time. Instead, the stereogram simply distorts.

### (g) Correspondence problem

**Figure 11.**
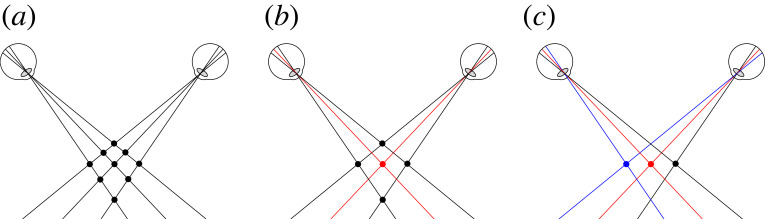
How rivalry eradication solves the correspondence problem. (Online version in colour.)

My rivalry eradication account also helps to simplify the correspondence problem (the process of matching points in the two eyes). On a triangulation account, any of the points in figure 11*a* would be possible [30] (the so-called ‘Keplerian array’), and this becomes a problem to be solved (e.g. by [30]'s smoothness constraint). By contrast, on my rivalry eradication account, because we're not trying to work out the position of points in the world, the problem of the ‘Keplerian array’ does not arise in the first place. Instead, on my rivalry eradication account, the visual system fuses whatever is on the two foveas (this is how the images are aligned and anchored before rivalry is resolved) (figure 11*b*). Then rivalry detection and eradication spreads out from the anchor at the fovea. So in figure 11, points on the left in both retinal images will be matched, and points on the right in both retinal images will be matched (figure 11*c*). This is an entirely retinal process, but figure 11*c* puts it in terms of the Keplerian array to illustrate how the percept that emerges is a flat row of dots.

### (h) Does V1 process 3D space?

My emphasis on resolving rivalry relative to the fovea is also critically important for resolving a fundamental tension in the neuroscience literature [27]. On the one hand, there is increasing evidence of highly cognitive processing in the primary visual cortex (V1) (which I review in [27]), from the integration of vision and location [31], to potentially the semantic category of sounds [32]. On the other hand, so far as stereo vision is concerned (the most basic and low-level aspect of 3D vision [24,33,34]), V1 is thought to be merely a preliminary feed-forward stage, with depth perception coming much later [35–37].

The fundamental question is whether V1 can account for our experience of stereo depth. Two arguments have been given against the V1 account. First, it's argued that our experience of stereo vision is primarily one of relative depth between points (which is what we are most sensitive to), whilst V1 processes absolute disparity (depth relative to the fixation plane) [38]. So [39] suggests that any plausible account ‘eliminates the fovea as a reference point’ and ‘avoids reference to any retinal landmark’. By contrast, on my account, that is exactly what we want to preserve, as the fovea provides the anchor by which to assess whether two points rival one another or not.

Another reason for suggesting that stereo depth isn't processed in V1 is that anticorrelated random-dot stereo images (where the points are white in one eye, and black in the other) fail to produce a depth percept even though they are still processed by V1 [40]. But if stereo depth is rivalry eradication, that's to be expected. The points are different colours, and so fusing them in depth isn't going to eradicate their rivalry. The best solution for the visual system is to simply let them rival one another as binocular lustre, which is what the literature reports.

For a broader discussion of the relationship between V1 and 3D visual experience, please see my neuroscience-focused companion piece to this article [27].

## 2. Perception/cognition distinction

### (a) Minimal theory of 3D vision

My rivalry eradication account of stereo vision constitutes a minimal theory of 3D vision. It decouples 3D vision from trying to estimate the properties of the world and can explain our experience of a random-dot stereogram viewed in darkness.

**Figure 12.**
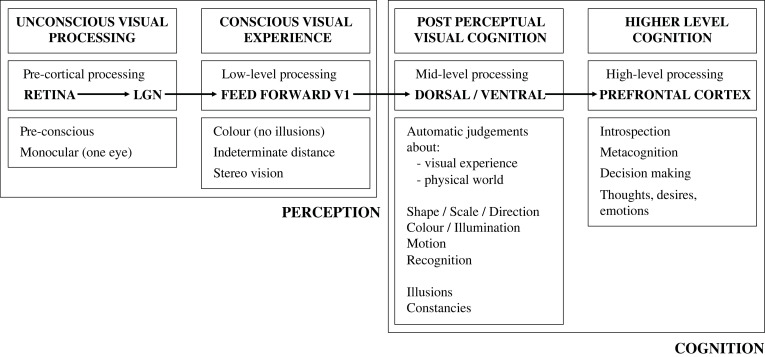
The perception/cognition distinction on my account. [41], Fig. 3, updated in light of [27]. It presents a low-level theory of visual consciousness [27,42], in contrast with mid-level theories (recurrent processing theory [43]; integrated information theory [44]) and higher-level theories (higher-order thought [45]; global workspace theory [46]). (LGN = lateral geniculate nucleus).

However, in my published work [27,41], I make a stronger claim, namely that this minimal theory of 3D vision is *all* that's involved in 3D vision. Figure 12 illustrates the structure of my account as it has been published in [27,41]. And the purpose of the remainder of this article is to explore what experimental strategies might help us differentiate between my minimal theory of 3D vision and traditional accounts.

### (b) Key distinction: stereo vision (perception) versus pictures (cognition)

We want a theory of real-world 3D vision, but the 3D vision literature risks giving us a theory of picture perception instead. The key problem is that vision science doesn't draw a sharp enough distinction between pictures and real-world 3D visual experience.

In [41, pp. 89–103] and [29,47], I argue that pictorial depth is merely cognitive. What do I mean by this? Think about a picture printed on a transparency and viewed in the real world. There's no sense in which the objects in the picture move off the surface of the transparency, either in front or behind it. They're literally stuck to the surface. So there's no real-world perceived depth. And I argue that the presence/absence of the visual experience of depth is exactly what marks the distinction between the perception/cognition of depth.

Contrast this with the alternative where pictures are just as much 3D vision as stereo vision. Here you're missing the fact that the separation between objects in depth is literally perceived in one context (stereo vision) but not in the other (pictures). Or, as I explain in [29], the fact that ‘while someone viewing a car and a photograph of a car may be able to identify the same object, they don't literally perceive the same 3D shape.’ By contrast, if you classify pictures as actual seeing 3D shape, then someone looking at a picture of a car just as equally sees the 3D shape of the car as someone looking at the car in real life. But that seems to miss exactly what's important about 3D vision. Instead, it's better to say that when we view a picture of a car, we recognize the car's 3D shape, even though we don't literally perceive it.

Unfortunately, contemporary vision science [48,49] argues that pictures:

‘…belie such simplistic, ‘cognitivist’ explanations, and current scientific research in visual perception rarely entertains them; seeking, rather, to understand picture perception phenomena from the viewpoint of the working of visual mechanisms (see [50–52])’. [49].

The two leading accounts of pictorial depth in the vision science literature treat it either as (i) a weak form of normal depth perception [53], or (ii) the perception of 3D shape [49,54–56]. Indeed, when I developed my purely cognitive account of pictorial depth in 2017 ([41, pp. 89–103]), I didn't know of any contemporary vision scientist who argued that we don't see depth in pictures (more recently, [57] has also come out in favour of a cognitive account).

The question that the remainder of this article poses is whether the remaining aspects of 3D vision, such as visual scale (size and distance estimation), visual shape, visual direction, and visual constancies, operate like stereo vision (perception) or pictures (cognition)? And I explore the experimental strategies that might help us to differentiate between these two accounts.

### (c) Other ways of thinking about the perception/cognition distinction

The way I draw the ‘perception’/‘cognition’ distinction doesn't necessarily fit with how the literature typically draws the distinction.

#### (i) Conscious deliberation

Some people associate ‘cognition’ with conscious deliberation and ‘perception’ with automatic judgements [58–60]. But I think that's a mistake. Take the example of reading [41, pp. 14, 97–99]. It's an automatic judgement, but we wouldn't want to equate it with perception. Indeed, controlling for eye movements, two people looking at words on a page will have the same visual experience even if one can read the words, but the other cannot^2^. So reading provides evidence of a third category—between perception and conscious deliberation—of automatic cognition, that needs to be recognized.

Reading also addresses another common criticism: the suggestion that on a cognitive account of pictures, viewers ‘have no impressions of pictorial space, but are only aware of planar patterns of colored patches’ [61]. When we read, we are aware of more than marks on a page. And yet this doesn't imply that we visually experience a ‘reading space’.

#### (ii) Higher-order cognition

When asking whether perception is ‘cognitively penetrable’, people are asking whether our perception is affected by our thoughts, desires, and emotions [62–64]. By contrast, on my account, this question is really about whether higher-level cognition ‘cognitively penetrates' automatic cognition (figure 12). For instance, as we will see below, I argue that the Müller–Lyer illusion is cognitive rather than perceptual [41, p. 63]. So, on my account of the Müller–Lyer illusion, (i) perception is veridical (we see the lines as having the same length), but (ii) automatic cognition is distorted (the illusion biases our judgement of our own visual experience), whilst (iii) higher-level cognition is veridical (we know the lines are the same length). So asking if the Müller–Lyer illusion is ‘cognitively penetrable’ is asking whether automatic cognition can be affected by higher-level knowledge. It would be like asking whether, if we know we are in France, this stops us from reading street signs in English?

For an illustration of the contrast between my position and Chaz Firestone's [62] position on the perception/cognition distinction, see [29,65,66].

### (d) Testing the perception/cognition distinction

It's often assumed that it's simply obvious whether something is perceptual or cognitive. Take one-eye (monocular) vision. Landy *et al.* [67, p. 396] insist: ‘when you close one eye the world does not suddenly become flat’. But the problem with this approach is that there are plenty of phenomena that are obvious at first blush that turn out to be false. For instance, high resolution in the periphery [68].

The point is, we do experiments because we believe that people can be mistaken about their visual experience through introspection. And on my account, visual cognition is automatic and involuntary, so we can't interpret our visual experience in an unbiased way. You can put this point in philosophical terms [69–71] by saying that people can have conscious visual experiences (phenomenal consciousness) that they are unable to access (access consciousness). But this skepticism of introspection is not new, and it is the very reason why psychophysics emerged as a discipline in the mid-19th-century.

So, the question then becomes ‘what experiments should we do?’ And the problem is that putting observers in the laboratory and asking them to judge their perceived depth, which is what most experiments do, is little better than naïve introspection. Instead, a truly psychophysical approach to 3D vision will look for direct comparisons between stimuli.

First, it will look for differences. So, if we manipulate a parameter, does it affect our visual experience in the relevant way? For instance, I argue that visual scale from stereo vision (the size that a scene is judged to be) is merely cognitive because if we make a direct comparison between one-eye (monocular) vision and two-eye (stereo) vision, there's no sense in which the scene either (a) changes in angular size, or (b) moves towards us or away from us, as we open and close one eye, and so there is no sense in which stereo vision actually changes visual scale (size and distance) in our visual experience.

Second, a truly psychophysical approach will look for commonalities, especially with a ‘ground truth’ or ‘gold standard’. For instance, monocular (one-eye) stereopsis is the claim that motion, perspective, and shading can produce 3D percepts equivalent to stereo vision, especially in synoptic viewing (where both eyes see the same image). In which case, as I discuss below, we should be able to make direct comparisons to stereo vision as a ‘ground truth’ or ‘gold standard’, by introducing points with disparity and seeing if they match the 3D depth from pictorial cues.

The following sections therefore attempt to operationalize the perception/cognition distinction through specific experimental predictions: ‘If phenomenon *x* is perceptual, we would expect ‘A’, but if it is merely cognitive, we would expect ‘B’.’ You might not agree with the claim (maybe a perceptual account could produce ‘B’?). Or you might think that the experiment, when tested, will go in the direction of perception (the experiment will give ‘A’). But the purpose of the remaining sections is to give my take on how the perception/cognition distinction is best operationalized in practice.

## 3. Two-stage theory of visual processing

In contrast to traditional accounts of 3D vision, which integrate all the depth cues together into a single coherent percept (at the level of visual experience), I argue for a two-stage theory of visual processing, where stereo vision resolves 3D structure at the level of visual experience (‘conscious visual experience’ in figure 12), whilst the other depth cues such as motion, perspective, and shading contribute (in addition to the perceived 3D structure from stereo) to our automatic 3D cognition or understanding of the scene (‘post-perceptual visual cognition’ in figure 12). On my account, stereo vision is a solution to a purely internal problem (rivalry eradication). But we link the minimal 3D percept that emerges from stereo vision to the world–through experience–at the level of cognition.

### (a) Internal (phenomenal) versus external (physical)

To emphasize, my minimal approach to 3D vision decouples 3D vision (stereo vision) from trying to estimate the properties of the world. As I argue in [41, pp. 74, 85], [27,72], 3D vision ‘operates *purely* at the level of *phenomenal geometry* and makes no claims about the *physical geometry* of the *physical world*’ [41, p.74]. Instead, it's cognition that links our 3D visual experience (something that's not about the world) to the world.^3^

### (b) Innate versus acquired

When it comes to the innateness of visual space, there is often a false dichotomy between ‘all’ (assuming an innate Euclidean model of space) vs. ‘nothing’. We inherited this false dichotomy from Kant [74] (all) vs. the British Empiricists (nothing) (Locke [75], Berkeley [76] and Hume [77]), and it continues to play out in the literature, both in animal navigation (O'Keefe & Nadel adopting a Kantian approach [78], Ch.1), and computer vision (the ‘all’ approach in ‘neural radiance fields' [79,80] vs. the ‘nothing’ approach of ‘neural scene representations' [23]).

**Figure 13.**
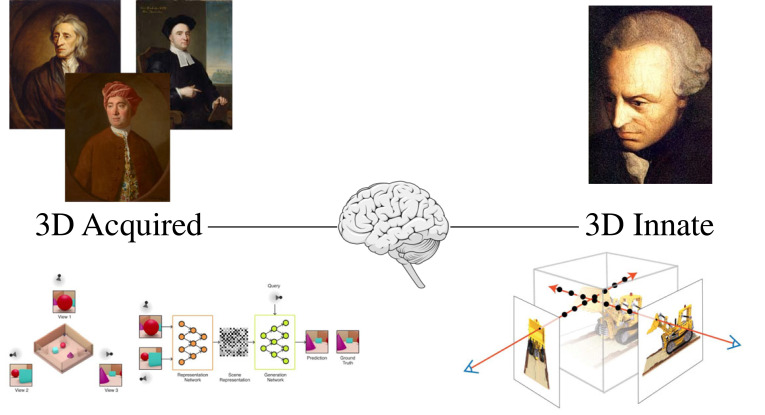
Is our experience of 3D space innate or acquired? Left: DeepMind's ‘Neural Scene Representation’ [23] follows Locke, Berkeley, and Hume in learning 3D space from scratch. Right: Neural Radiance Fields' (NeRFs) [79] follow Kant in assuming ‘geometry awareness’ (3D space) from the outset. Middle: I place the human brain halfway between these two extremes, so depth from disparity is innate, but 3D space has to be learnt. Images from [23] and [79] © authors. (Online version in colour.)

By contrast, I place the human brain halfway between these two extremes (figure 13) and suggest a partial innateness. First, I've argued that stereo vision with zero disparity (and, what is the same thing on my account, one-eye vision) consists of 2D + 1D: a 2D phenomenal plane, seen at a single fixed phenomenal distance (1D). For instance, in [41, pp. 134–6], I argue:

‘…the plane of fixation *always* corresponds to a *single fixed phenomenal depth*, and so whatever is being fixated upon, whether it is near or far, is seen as being at that *single fixed phenomenal depth*’.

Second, depth from disparity (3D vision) also appears to be innate. Once the eyes are aligned, stereo vision can snap into place even after decades of monocular (one-eye) vision [81–83]. By contrast, visual scale and pictorial cues [84,85], and even depth from motion ([82, pp. 102–3], cf. [85]), requires considerable perceptual learning. This suggests a distinction between perceptual depth cues (stereo), which are innate, vs. cognitive depth cues (motion, perspective, shading) that are acquired through experience.

But third, even if 3D vision from stereo is innate, it's important to understand how minimal this 3D percept is, and what else has to be learnt through experience before we can talk about ‘3D space’. The retinal image (and so, on my account, our minimal 3D percept) changes with every bodily movement and every eye movement. So we have to learn (at the cognitive level) to link changes in our 3D percept to our motor commands (bodily movements and eye movements) so we can build-up the concept of a 3D space in which we act. So ‘3D space’ is a cognitive (not visual) space.

This suggests a very different approach to perceptual learning than currently employed in deep neural networks (figure 13) (for a review, see the Introduction article to this issue [86]). On my account the input to perceptual learning would be depth from disparity (so something more than the 2D image input in [23]), but we would still have to learn the concept of 3D space as a space in which we move, and which contains 3D objects that persist across time (so something less than the innate 'geometry awareness' of [79]).

### (c) Individual differences

Read [87, p. 117] highlights the ‘key assumption of much psychophysics: that it examines the most basic, fundamental aspects of human perception, common to all normally functioning humans, rather than more subtle aspects of human experience that might fluctuate within or between individuals’. By contrast, pervasive individual differences seem likely to reflect cognitive rather than perceptual mechanisms under my account. For example, depth from shading [88].

## 4. Visual scale

### (a) Stereo vision divorced from physical size/distance

Visual scale is the perception of the physical size and distance of objects and scenes. I've argued that in stereo vision (figure 1), vergence and vertical disparities don't provide us with the distance of the fixation plane. Instead, the fixation plane is experienced at a single fixed ‘phenomenal distance’, no matter what its physical distance is ([41, pp. 134–6], [89,90]).

Note that this single fixed ‘phenomenal distance’ is not Gogel & Tietz's [91] ‘specific distance tendency’, the assumption that, in the absence of distance cues, objects default to a physical distance of 2–3 m. I find no evidence for this in my experimental work (see the raw data of [11], fig. 5).

Instead, this single fixed ‘phenomenal distance’ corresponds to all potential physical distances. There's an intuitive appeal to this account [89]. The fact that we see a movie screen at a single fixed phenomenal distance, and yet a movie can transition from a wide-panning shot to a close-up without being jarring, suggests that attributing different scales to a single fixed phenomenal distance is something that comes naturally for humans.

### (b) Physical size/distance from stereo is merely cognitive

But how do we square this with the fact that stereo vision dominates our everyday experience of visual scale? As Helmholtz demonstrated in 1857 [92,93], if you use mirrors to artificially increase the distance between the eyes (‘telestereoscopic’ viewing) and then look at the world, you get the impression that we are ‘not looking at the natural landscape itself, but a very exquisite and exact model of it, reduced in scale’ [94, p. 312]. This is despite the fact that we have every other distance cue giving us veridical distance. So whatever explains why we see the world as the wrong size in telestereoscopic viewing, must also explain why we see the world as the right size in normal viewing.

Traditionally telestereoscopic viewing has been attributed to vergence and vertical disparities [94–96], but I've argued against these as absolute distance cues. Instead, in [90, p. 26], I argue that stereo vision's ability to affect the scale of a scene relies on ‘a cognitive association between (a) vivid stereo depth and (b) closer distances (reflecting our experience of an environment where disparity falls-off with distance^2^)’. See also [12, pp. 17–22].

**Figure 14.**
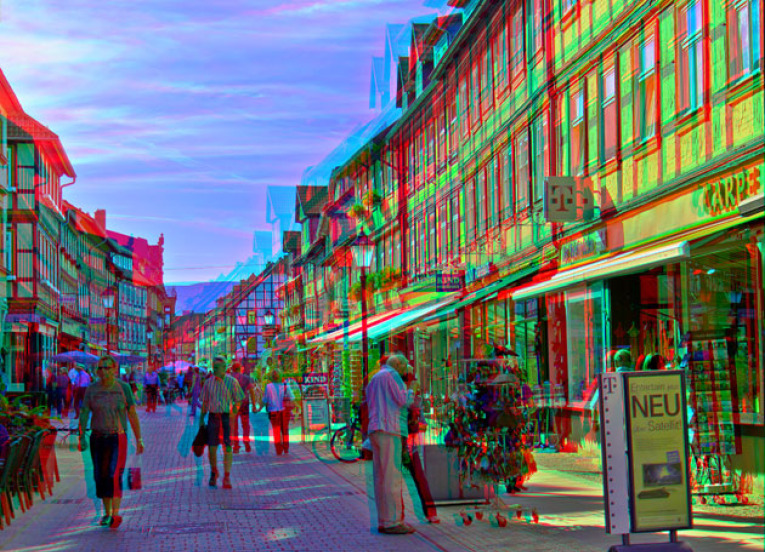
An example of telestereoscopic viewing where the world appears miniature. ‘Wernigerode Boulevard’ (2011) by Sascha Becher. © Sascha Becher. https://www.flickr.com/photos/stereotron/6597314627/in/album-72157612377392630/ Note that this effect isn't limited to stereograms, and is actually experienced in the real world when mirrors are used to artificially increase the distance between the eyes. (Online version in colour.)

It's important to emphasize at the outset that this is a purely cognitive mechanism. What do I mean by this? View figure 14 with 3D glasses. With one eye closed, it looks like a normal street scene. With both eyes open, it looks miniature. But what is the difference in our visual experience between these two conditions? Clearly, with both eyes open, the scene has accentuated stereo depth. But fixate on an object and open and close one eye. Does the object appear to move towards you when you open both eyes? No. Does the object appear to reduce in retinal size when you open both eyes? No. So there is no sense in which stereo vision is changing your visual experience of size and distance. And yet the scene seems miniature. But all this can be, then, is merely a cognitive association between accentuated stereo depth and reduced size.

**Figure 15.**
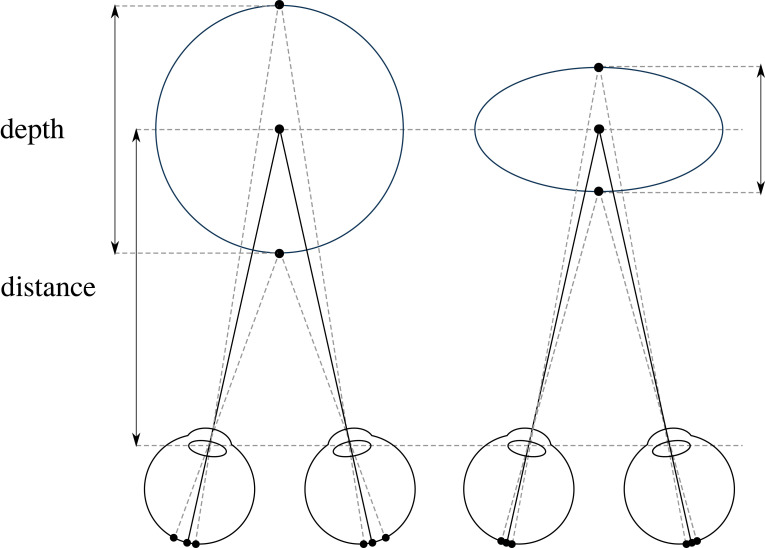
Manipulating horizontal disparities affects the depth, but not the distance, of objects.

Indeed, this insight almost seems inevitable if, like me, you seek to ground visual scale in horizontal disparities, since horizontal disparities are (rightly) thought to only affect the depth of the object being viewed, not its distance from the observer (figure 15).

### (c) Inverting the relationship between depth and distance

Horizontal disparities therefore seem a poor candidate for an absolute distance cue. But the cognitive association between (i) vivid stereo depth, and (ii) closer distances, squares this circle.

On traditional accounts, perceived stereo depth is a function of disparity scaled by distance (distance → depth) [18,97,98]. By contrast, on my account, perceived stereo depth is simply a function of disparity alone, so objects will be perceived as having more depth the closer they are^4^. And I argue we learn this relationship between accentuated stereo depth and nearer distances. So this relationship (depth → distance) explains why we experience figure 14 as small. We only ever experience the accentuated stereo depth in figure 14 when we look at a small scene up close rather than a large scene far away.

### (d) All distance cues are merely cognitive

So far, I have argued that the association between (i) vivid stereo depth and (ii) closer distances is merely a cognitive (rather than perceptual) cue to visual scale. But in published work ([41, pp. 118–136], [11,12,89,90]), I argue that all distance cues function in this way, and that our experience of visual scale (the absolute size and distance of objects and scenes) is merely cognitive.

How should this be tested? Take familiar size. Imagine we view a luminous patch in darkness with all the other distance cues removed. Then we replace the luminous patch with a familiar object that is the same size and the same distance as the luminous patch. Does the familiar object appear to move towards us or away from us (indicating a perceptual mechanism)? Or does the familiar object seem to be located at the same distance, only now we have a better idea of what that distance is (indicating a merely cognitive mechanism)?

There's strong suggestive evidence that visual scale is merely cognitive. First, stereo vision dominates binocular (two-eye) visual scale, and familiarity (object/scene recognition) dominates monocular (one-eye) visual scale. Second, I've already argued that visual scale from stereo vision is merely cognitive. Third, it is already widely accepted that visual scale from familiarity (object/scene recognition) is merely cognitive as well. The suggestion in the literature, from Descartes [2] to the present, is that familiar size only affects the distance at which objects are judged to be, but not the distance at which they are perceived to be [54,99–110]; although see [111] for an alternative.

The only step in the argument that still needs to be justified is the suggestion that familiarity (object/scene recognition) dominates monocular (one-eye) visual scale. To convince you of this, think about how much of our daily lives are conducted on screens, but how rarely we experience ambiguity as to scale. We view photos, watch movies, and teleconference with one another without traditional monocular (one-eye) depth cues (accommodation, motion parallax, and the ground plane^5^). But our impression of visual scale in all these contexts is effortless and rarely in error. And the only thing that could provide size and distance information in this context is our visual recognition of scenes and objects.

Now contrast familiarity with other alleged monocular (one-eye) distance cues, which I argue [11,12,90] are largely ineffective and/or very limited in application:

#### (i) Accommodation

The closer an object is, the more the eye has to increase its power to bring the object into focus. But [112] find accommodation provides ‘no functionally useful metric distance information’, and I also find no benefit when it's tested alongside vergence [11].

#### (ii) Motion parallax

[113] concludes that ‘there is no empirical evidence that providing motion parallax improves distance perception in virtual environments' and [114–118] find very little benefit from motion of the observer.

#### (iii) Defocus blur

Tilt-shift miniaturization exploits extreme defocus blur across the visual field [119,120]. But as [119] demonstrate, this blur is rarely evident in everyday viewing.

#### (iv) Ground plane

How far along the ground an object is [121] and/or how high it is in our visual field [122,123] are often taken to be important absolute distance cues. But first, we have to be careful to distinguish the ground plane from other distance cues, such as the familiarity of the scene. Testing in fields and corridors does not do this, and there's been little investigation in virtual reality of Gibson's ideal of the ground plane as an abstract texture gradient viewed by a monocular (one-eye) observer [124–127]. Second, an extended ground plane is rarely a defining feature of most everyday visual experience. Third, the ground plane cannot help us in reaching and grasping, the one context where we really need accurate distance information.

In conclusion, our understanding of visual scale has fossilized for half a century. Contemporary discussions [128] and textbooks [129] typically cite Cutting & Vishton from 1995 [130], but Cutting & Vishton's famous diagram of the different distance cues and their range is simply a redrawing of an earlier diagram by Nagata from 1977 [131,132]. As Cutting & Vishton [130] note: ‘Perhaps the most curious fact about psychological approaches to the study of layout is that its history is little more than a plenum of lists.’

By contrast, I've argued that the traditional list needs to be challenged [11,12,90]. First, (i) vergence, (ii) vertical disparities, (iii) accommodation, (iv) motion parallax, (v) defocus blur, and even (vi) the ground plane, have been shown to be either largely ineffective as absolute distance cues and/or very limited in application. Second, all this leaves us with is (vii) familiarity (object/scene recognition) and (viii) my new distance cue, stereo depth (primarily from horizontal disparities). Stereo depth seems to dominate binocular (two-eye) visual scale, whilst familiarity seems to dominate monocular (one-eye) visual scale. Importantly, I've argued that both of these cues are merely cognitive in nature, challenging the idea that we directly perceive size and distance.

## 5. Visual shape

We want a theory of real-world 3D vision, but the 3D vision literature risks giving us a theory of picture perception instead. As I note in [29], ‘while someone viewing a car and a photograph of a car may be able to identify the same object, they don't literally perceive the same 3D shape’.

The problem is that we don't have a good theory of how to differentiate real-world 3D vision (perception) from the pictorial depiction of depth (cognition). But without this, we are unable to differentiate the viewing conditions in which we truly perceive 3D depth.

### (a) Cue integration^6^

Cue integration claims that all the different depth cues (disparity, perspective, shading, and motion) are integrated into a single coherent percept in real-world 3D vision [67,133–136]. To support this claim, there's been a vast literature over the past 20 years that appears to demonstrate that perspective, shading, and motion can all be traded off against stereo vision [137]. Notably, [135] suggest that stimuli made up of different combinations of depth cues can be perceptually indistinguishable from one another.

But in order to test if this claim is really true, we need to test it in real-world conditions. By contrast, as I argue in [41], Ch.2, the literature effectively cheats by separating the stimuli in space and time, something that we almost never experience in real-world viewing.

#### (i) Separation in time

First, stimuli with different combinations of stereo and texture are only indistinguishable from one another in [135] because the stimuli are shown with time intervals between them. This changes the task from a perceptual task to a working memory task. So first, time intervals between stimuli need to be eradicated.

#### (ii) Separation in space

Second, why think the successive presentation of stimuli is the right test? In everyday viewing, we see objects alongside one another. If cue integration does not persist in the presence of other objects, it cannot be a plausible theory of real-world 3D vision. In [41], Ch.2, I argue that we should be able to introduce stereo bars that mark out the fronto-parallel plane, and see if the cue integration stimuli from [138] really look slanted against it? In [41, p.51], fig. 4, I mock this up, and no slant from pictorial cues is apparent.

### (b) Monocular (one-eye) stereopsis^7^

Monocular stereopsis' is the claim that if you view pictures and movies with one eye through a pinhole (to remove cues to the screen's flatness) the depth cues in the pictures and movies will transform themselves into real-world 3D vision [53,139]. So someone looking at a picture of a car with one eye, and someone looking at a real car with two eyes, can really have the same 3D experience.

But again, the literature never tests this hypothesis in a way that pitches stereo cues against pictorial and movies cues. If I really have the same 3D percept looking at a picture of a car in this way, I should be able to replace it with a stereo car and not notice the difference.

But ‘monocular stereopsis' isn't just a claim about pictures and movies, it's also a claim about our monocular (one-eye) vision of the real world.

I fit monocular (one-eye) vision into my account by thinking of monocular (one-eye) vision as two-eye (stereo) vision where the eyes have exactly the same retinal image (zero disparity). This occurs naturally when looking at the horizon and can be induced artificially by synoptic viewing (showing the same image to the two eyes) [132].

But synoptic viewing also provides us with a true test of ‘monocular stereopsis'. First, as I've already alluded, we should be able to replace a pictorial 3D object with a stereo 3D object, and not notice any change in depth. Second, if monocular stereopsis really is equivalent to stereo vision, we should be able to measure it using stereo vision, by introducing points with disparity to mark out the perceived depth from pictorial and movie cues. By contrast, if depth from pictorial and movie cues can't be measured in this way, then we're back to treating pictorial and movie cues merely as picture perception rather than real-world 3D vision [41, pp. 103–110].

These experiments haven't been done. However, I would argue that three existing experiments already demonstrate that (i) what observers judge the perceived depth from pictorial and motion cues to be, and (ii) what the perceived depth from pictorial and motion cues actually is, are two very different things. In these experiments, participants think they see *x* amount of depth. But when we show them *x* amount of depth from stereo, it becomes immediately apparent that they were actually experiencing much less depth, and I would argue no depth at all.

#### (i) Motion (kinetic depth effect/motion parallax)

Participants in [140] (discussed in [41, pp. 38, 46–8], [141]), judged a monocular (one-eye) motion-only stimulus to have 15% more depth than a binocular (two-eye) stereo + motion stimulus, implying that motion (kinetic depth effect) by itself had a greater effect on perceived depth than stereo. But if that's true, then when participants close one eye whilst watching the stereo + motion display (turning it back into the motion-only stimulus), they should see an increase in depth. But that's not what [140] found. Instead, all the participants reported seeing a marked reduction in depth. This suggests that when participants reported the perceived depth from motion, they mistook (and reported) the depicted depth on the screen, rather than their actually perceived depth.

#### (ii) Motion (looming/optic flow)

Participants in [142] (discussed in [141]) were shown two motion in depth stimuli at the same time: (i) a looming stimulus and (ii) a point with disparity. The participants judged the stimuli to have roughly the same absolute motion in depth (looming = 15.2cm, disparity = 16.4cm; observers ‘confirmed … they appeared to move a similar amount in depth’). Yet, despite these similar absolute judgements, participants still reported seeing considerable relative depth between the stereo and looming stimuli. Again, when reporting the perceived motion of the looming stimulus, participants appear to be mistaking (and reporting) the depicted motion for their actually perceived motion in depth.

#### (iii) Pictorial cues (perspective/shading)

The participants in [143] (discussed in [41, p.108], [141]) judged the depth of pictures in the presence and absence of stereo cues. Pictorial cues could account for 96.6% of the variance in the reported depth, with stereo vision accounting for just 1.2% of the variance. As [143] admit: ‘Given the immediate impression of the stimuli, it is perhaps surprising that we did not find a larger scaling effect’. Again, it appears participants are mistaking (and reporting) depicted depth for their actually perceived depth.

In conclusion, ‘monocular stereopsis' is an extremely strong claim: that motion and pictorial cues produce real-world depth on a par with stereo vision once surface cues have been removed. But the only appropriate test is a direct comparison with stereo depth. When such comparisons have been made, pictorial and motion cues have failed this test.

What are the implications? There's an old debate on whether monocular (one-eye) vision is flat. In the mid-20th-century, both Ogle [144] and Julesz [24] appeared inclined to say that it was. More recently, this position has been advocated by those who have recovered [81,82] or lost [145–148] stereo vision later in life. In my book [41] I also argued for this position so far as pictorial and motion cues were concerned (cf. defocus blur^8^), and would now [27] argue that all monocular (one-eye) vision is flat in the same way that pictorial depth is flat.

## 6. Visual direction

### (a) Vision as retinotopic

**Figure 16.**
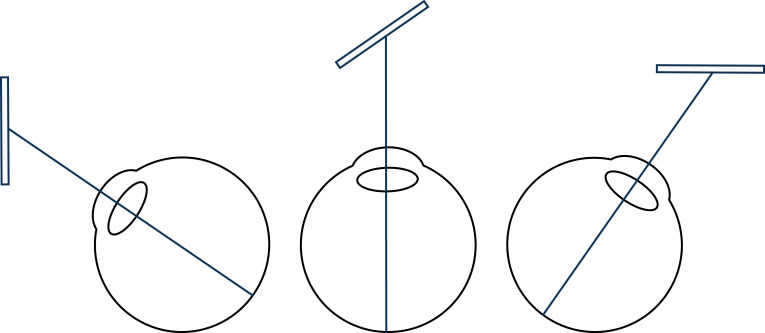
Different combinations of visual direction and surface slants give rise to the same retinal image, so visual direction is thought to be more than retinal direction.

Visual direction is generally thought to be more than retinal direction. For instance, the three different eye positions in figure 16 have the same retinal image, but are thought to produce very different visual experiences. The same literature also claims that eye movements affect the perceived size and distance of objects (see single-cell recordings [153–157], and changes in after-images with eye movements [10,151,152]). So, on orthodox triangulation accounts, visual distance and visual direction are thought to be combined so that visual signals are ‘dedicated to certain volumes of visual space’ [156].

By contrast, it's exactly for this reason that in [27,90] I insist that vision should be thought of in retinotopic, rather than in head-centric, body-centric, or world-centric terms. Since my experimental work has found no evidence of eye movements affecting perceived size or distance [11,12], we should be skeptical of the suggestion that eye movements affect perceived direction, especially since the very same experimental paradigms were previously used to (falsely) suggest that eye movements affect perceived size and distance.

Interestingly, keeping vision in retinotopic terms might also help us to explain the rubber hand illusion [158]. If the spatial location of vision (the rubber hand) and touch (the real hand) were in a common body-centric coordinate frame, it would be hard to see how such distant spatial locations could be integrated. By contrast, if vision is kept in retinotopic terms, and two further cognitive steps are required to convert vision into head-centric, then body-centric terms, the spatial slippage required to integrate these two locations becomes easier to explain.

### (b) Visual stability

Since the eyes typically move three times a second, it's suggested that to make the world seem stable, a perceptual mechanism is required to cancel out the motion of the world on the retina caused by eye movements [159–161]. ‘Clearly, extraretinal signals are important for visual stability’ [162]. However, if, as I argue, vision remains in retinotopic terms, then the mechanisms underpinning visual stability must be merely cognitive (post-perceptual) in nature [27, p. 6] since they can't operate at the level of our visual experience, which remains retinotopic.

Supporting this hypothesis is the fact that the perceptual mechanism commonly suggested for visual stability is simply too slow. In [151] an after-image was induced, and participants were then asked to move their eyes in darkness. Participants reported seeing the after-image move, even though the retinal image remained fixed, which was interpreted to be an indication of a perceptual translation from retinal to head-centric coordinates [151]. However, the reported shift in direction occurred 0.6–0.8 s after the eye movements had been made, which is much too slow to be of use for visual stability when 3 + eye movements are made every second.

### (c) Visual field and visual direction

The ‘visual field’, as famously articulated by Ernst Mach [163], is a description of our monocular (one-eye) visual experience. As Gibson [127, p.114] notes, ‘the *visual field* means a kind of introspective experience … It is the momentary patchwork of visual sensations.’ On my account, it can be thought of as our visual experience of the retinal image at an indeterminate distance (2D + 1D, discussed above in Section 3(b)).

The visual field has two dimensions: a *y*-axis height and an *x*-axis width, so we talk about ‘height in the visual field’ and ‘width in the visual field’. But note that our visual experience (of the visual field) doesn't convey visual direction. The reason is that the same visual experience (the same visual field) could have been produced by two very different projections—Perspective Projection and Orthographic Projection—with two very different models of visual direction.

**Figure 17.**
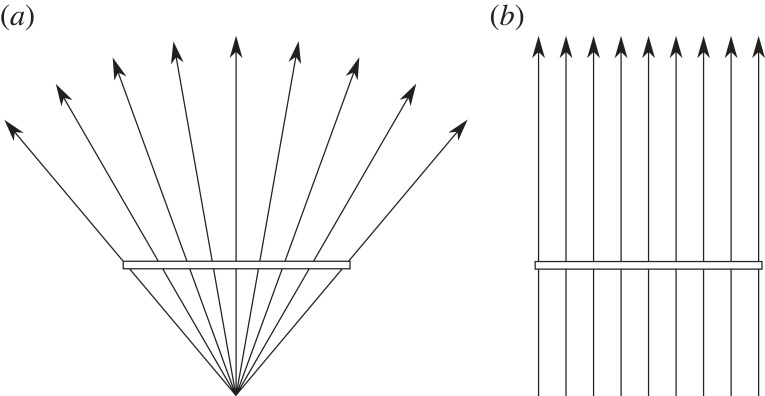
Visual direction according to Perspective Projection (*a*) and Orthographic Projection (*b*), where the rectangle marks the visual field. (Online version in colour.)

In reality, the retinal image is the product of Perspective Projection, which is why we see the world in perspective. On this account, visual direction is angular (figure 17*a*). By contrast, the retinal image could have been produced by Orthographic Projection (using a telecentric lens), where the world is not subject to the laws of perspective. In which case, visual direction is parallel (figure 17*b*). So how we map from our visual experience (the visual field) to visual direction requires a further, cognitive (purely associative), step that determines between these two possibilities.

Orthographic projection produces a Euclidean model of visual space, where parallel lines remain parallel. By contrast, Perspective projection distorts visual space: space expands with distance, so parallel lines seem to converge. This highlights a key tension in our cognition of visual space. On the one hand, we want to think of visual space as a Euclidean mapping of physical space, so parallel lines ought to be parallel, leading to the accusation that in vision we experience under-constancy. On the other hand, we've internalized an angular model of visual space (where visual space expands with distance) when we (i) point at objects in the world (angular visual direction) or (ii) use distance to estimate physical size (angular visual size). These two models of visual space are fundamentally irreconcilable.

### (d) Visual constancies and visual direction

#### (i) Size constancy

Size constancy is the claim that vision ought to correct for perspective projection in the retinal image, so that parallel lines are seen as parallel, and equally sized physical objects have the same perceived angular size no matter their distance [164,165].

But on an angular model of visual direction, parallel lines *do* converge *in angular terms* with distance and objects *do* get smaller *in angular* *size* with distance. So, if you think of visual direction and visual size in angular terms, there is no failure of size constancy to correct for.

This puts size constancy in conflict with Emmert's Law [166], in contrast with most accounts in the literature that equate Emmert's Law with size constancy [152]. In Emmert's Law, we attribute different physical sizes to objects based on their distance and angular size. So Emmert's Law is a very straightforward application of the angular direction model. By contrast, size constancy demands that the visual system actively distorts perceived angular size, partially undoing perspective projection, so that objects shrink less in angular size with distance, bringing vision closer to orthographic projection.

#### (ii) Depth constancy

The tension between thinking of vision in angular terms versus Euclidean terms also arises in depth constancy. Depth constancy is the claim that we should see objects as having the same perceived 3D shape with distance. And the fact that objects appear to get flatter with distance [98] is seen as a failure of depth constancy [167].

But again, in angular terms, the perceived *z*-axis depth of an object really *does* fall-off much faster (with distance squared) than its *x*-axis and *y*-axis angular size, so the object should be perceived as flatter with distance. This point is easier to grasp if we don't think about 3D shape, but instead just think about two points, one on the front and one on the back of the object. No matter the distance, the disparity on the retina accurately reflects the angular visual direction of those two points given the position of the eyes. So, on an angular direction model, there is no failure of depth constancy that needs correcting for.

### (e) Visual constancies and visual experience

The literature claims vision incorporates size constancy, depth constancy, and shape constancy, into our visual experience. But many of these claims are open to challenge.

#### (i) Size constancy

**Figure 18.**
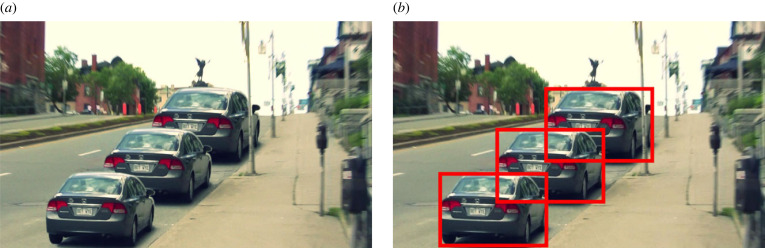
(*a*) The same car pasted three times in image. ‘Copy-paste, no resize’ by Alex Blouin (https://www.reddit.com/user/weip). © Alex Blouin. Original source: https://imgur.com/WBAzkuI (*b*) My addition of bounding boxes in [27,169]. The presence of the bounding boxes does not seem to seriously affect the illusion (compare the far cars in (*a*) and (*b*), and the near cars in (*a*) and (*b*)). (Online version in colour.)

Does the visual system change the perceived angular size of objects with distance? Both ‘vergence size constancy’ [10] and ‘pictorial size constancy’ [168] (figure 18*a*) say so. But I find no evidence of ‘vergence size constancy’ in [12], and in [27,169], I argue that ‘pictorial size constancy’ is a merely cognitive effect.

The suggestion in the literature is that pictorial size constancy is due to neurons in the retinal map in the primary visual cortex (V1) redrawing their boundaries in response to pictorial cues [168,170–172]. In computer graphics, this is known as ‘image warping’. But if this is correct, then every object in the same region of the 2D image should be equally distorted. However, this is not what we experience, since the bounding boxes in figure 18*b* are much less distorted than the cars, even though they occupy the exact same region of the image [27,169].

Now it's important to understand that image warping and pictorial size constancy are exactly the same claim. Pictorial size constancy [168] claims that in figure 18*a* the 2D angular size of the cars is distorted *in the image*. What's startling about figure 18*a*, and what leads people to reach for their rulers, isn't that we judge the cars to have different 3D physical sizes (Emmert's Law), but that we judge them to have different 2D angular sizes *in the image*, even though they have exactly the same 2D angular size *in the image*.

So the argument against image warping becomes a general argument against pictorial size constancy being perceptual rather than cognitive. If the 2D angular size of the cars was perceptually distorted in figure 18*b*, then so too would be the 2D angular size of the bounding boxes. But since there is no distortion of the perceived 2D angular size of the bounding boxes, there can't be any distortion of perceived 2D angular size of the cars. On an image warping account, and (what is the same thing) a size constancy account, they stand and fall together.

#### (ii) Depth constancy

In Section 1, I argued that stereo vision isn't trying to compensate for the fall-off of disparities with distance, and so objects appear to get flatter with distance. However, how do I explain the partial depth constancy reported in the literature [98,167]? Again, I argue it's a purely cognitive compensatory mechanism. We're used to objects getting flatter with distance, so if you ask me to set the depth of a sphere at 5 m, and I know it is at 5 m, I will set it flatter than if you asked me to set the depth of a sphere at 1 m.

There's an analogy here with size constancy. You're used to seeing parallel lines converging with distance. So if I ask you to draw parallel train tracks, you'll likely draw them as converging. But it would be a mistake to infer that you must therefore see the converging train tracks you draw as parallel in your visual experience.

#### (iii) Shape constancy

Thouless [173,174] reports that stereo vision undoes the rotation of objects to make them more fronto-parallel, reducing their aspect ratio. But again we can use bounding boxes to show this isn't true. The borders of figure 10 are intentionally clipped so that they fit the *x*-axis and *y*-axis angular size of the retinal image. Viewed with 3D glasses, you'll see the stereo percept fits perfectly inside the bounding box, so its aspect ratio remains the same.

## 7. Visual illusions

Do visual illusions affect our visual experience? Or do they merely affect our ability to accurately judge our veridical (non-illusory) visual experience? In [41, pp. 57–63], [169] and [27], I argue for the latter, purely cognitive, interpretation of illusions, and so prefer to label them ‘delusions’ rather than ‘illusions’.

There are some interesting parallels here with Goodale & Milner's [175–178]'s suggestion that action is immune to illusions. My way of framing this point is that action is often (but not always) a more accurate guide of our visual experience than our biased introspection.

### (a) Ponzo illusion

But how do we know if visual illusions affect our visual experience or not? Well, I've already developed this argument in the context of one illusion, the Ponzo illusion. Figure 18 is an instance of the Ponzo illusion [172], but I've already argued that 2D angular size is not perceptually distorted in figure 18.

### (b) Müller–Lyer illusion

**Figure 19.**
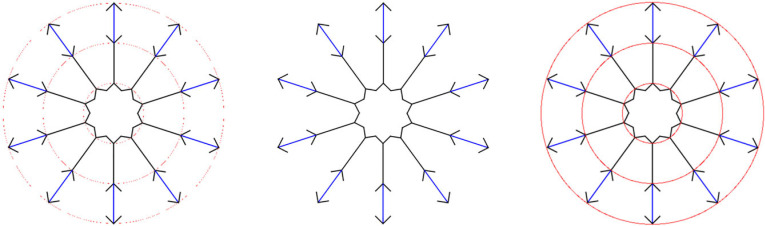
Gianni Sarcone's dynamic Müller–Lyer illusion (CC BY-SA 4.0): https://commons.wikimedia.org/wiki/File:Sarcone%E2%80%99s_PulsaOng_Star_ (Dynamic_M%C3%BCller-Lyer_illusion).gif, with bounding circles added in [169,179]. Animated gif: https://osf.io/mdaqt/. (Online version in colour.)

In [169], I apply the same approach to Sarcone's dynamic Müller–Lyer illusion (figure 19). The question is, what happens when we add static circles to the dynamic Müller–Lyer? For some, including one reviewer, the circles extinguish the illusion. For others, including the author, the illusion persists. Try it for yourself: https://osf.io/mdaqt. If the illusion persists in spite of the circles, then we can make an argument similar to figure 17. Since the circles don't appear to move, and since the circles and the perceived lengths of the lines are co-extensive in our visual experience, the perceived lengths of the lines cannot be changing either.

### (c) Hollow-face illusion and reverspectives

3D visual illusions such as the hollow-face illusion (where shading appears to invert disparity) [180–182] and reverspectives (where perspective appears to invert disparity) [183–185] challenge my suggestion that stereo vision and pictorial cues are processed at different levels. The hollow-face illusion and reverspectives appear to involve a *global* inversion of visual space, explaining why the 3D geometry of the face or scene is preserved, albeit inverted.

However, in my book [41, pp. 57–63], I present a new variation of the hollow-face illusion that challenges the global inversion of visual space. The question is what happens when we put physical objects into the hollow of the hollow-face? The hollow of the hollow-face is ‘impossible’ space so far as the illusion is concerned. It shouldn't exist, because it's now behind the surface of the face, not in front of it. So what happens when we put physical objects into this ‘impossible’ space? Interestingly, the illusion seems to persist. And yet, the true depth relationship between the physical object and the hollow-face can still be seen. The object is seen where it (and the hollow) truly are: in front of the nose, and behind the ears, of the hollow-face. But that shouldn't happen on a global depth inversion account.

The mask is supposed to be inverted (distance_ears_ > distance_nose_), but with the presence of another object we can directly perceive that (distance_nose_ > distance_object_ > distance_ears_). And in what sense can we really claim that we see the hollow-face as inverted in depth if we continue to see the veridical depth relations (distance_nose_ > distance_ears_) between points on the mask? Going forwards, I argue in [41, p. 58] that we could make really fine-grained judgements by filling the hollow of the hollow-face with points defined by disparity.

### (d) Light and colour constancies

**Figure 20.**
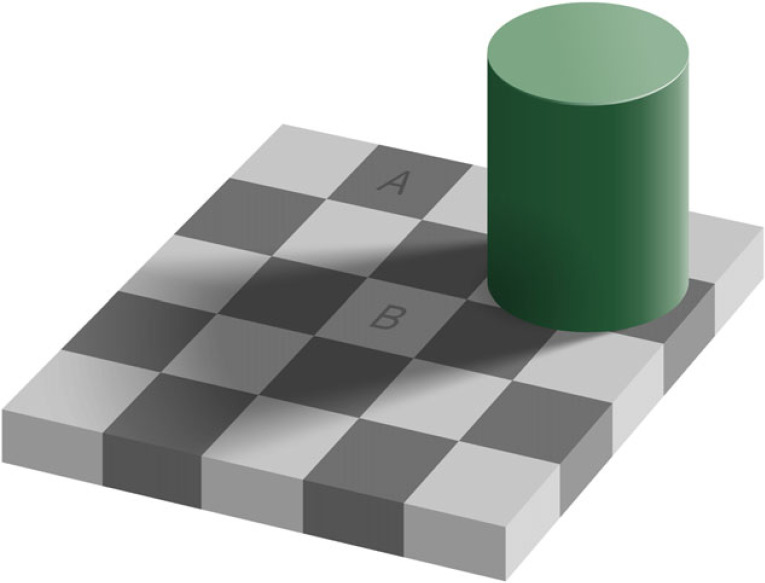
Adelson's Checker-Shadow Illusion. The squares A and B are the same shade of grey. (CC BY-SA 4.0). https://en.wikipedia.org/wiki/File:Checker_shadow_illusion.svg. (Online version in colour.)

In [27], I apply a similar argument to light [186] and colour [187] constancies. Squares A and B in figure 20 are physically the same shade of grey in the image, yet everyone would say the illusion changes our visual experience so we see different shades of grey. By contrast, I would argue that we see the same shade of grey in A and B, but that the pictorial cues bias our ability to accurately judge what our own visual experience of A and B is. But how do we know if light and colour constancies affect our actual visual experience or merely our ability to judge our visual experience?

I argue that we should take advantage of the fact that colours and shades have absolute values. We can find the absolute value of the ‘light’ patch by showing the illusion, and then (without interval) replacing it with a full-screen of grey, until we find a match. And we can do the same for the ‘dark’ patch. If the two absolute values turn out to be the same shade of grey, I would argue that we should conclude that observers are visually experiencing the same shade of grey, and the illusion only affects our ability to judge our visual experience.

If this turns out to be true, the deeper conceptual point would be that illumination itself (lighting in the environment) is not directly conveyed by visual experience, and that all that is conveyed instead are simply patches of retinal illuminance.

## 8. Multi-sensory integration

If you view an after-image of your hand in darkness, and move your physical hand closer or further away from your face, the after-image of your hand will appear to shrink or grow in size. This phenomenon, known as the ‘Taylor illusion’ [10,188–190], has led to the suggestion that vision ‘relies on multimodal signals' [10] and that ‘visual consciousness is shaped by the body’ [191]. But does multi-sensory integration really affect vision? The leading account of the Taylor illusion is that the vast majority of its effect is due to vergence, and only a negligible amount is due to the hand movements [10]. But my experimental work has shown that vergence has no effect on perceived size [12], suggesting that the vergence component of the ‘Taylor illusion’ is merely cognitive. If a merely cognitive effect can account for the major (vergence) component of the ‘Taylor illusion’, then it's highly likely that a merely cognitive effect can also account for the minor (multi-sensory) component as well. So I'm skeptical of the ‘Taylor illusion’ as telling us anything about visual perception.

Instead, multi-sensory integration appears to operate at the level of cognition. Take Hillis *et al.*'s [135] finding that estimates of surface slant from vision and touch are not fused together (in ‘Mandatory Fusion Within, but Not Between, Senses’). You can attend to the visual estimate of slant. And you can attend to the touch estimate of slant. And yet, when asked to estimate surface slant, subjects will typically provide an average between the two. But where does this average exist? It doesn't exist in vision, which reports the visual estimate. And it doesn't exist in touch, which reports the touch estimate. So there is no specific perceptual experience (or qualia) (neither vision, nor touch) that reports the average multi-sensory estimate [141].

## 9. Conclusion

This article argues for a two-stage theory of 3D vision, where our purely internal ‘minimal’ 3D percept (from stereo vision) is linked to the world through cognition. This leads to a decisive break with a number of traditions. First, it rejects Kepler and Descartes' triangulation model of vision, and argues that it was a historical mistake that we had this model for 200 years before we realized that retinal disparities were responsible for stereo vision. Second, it rejects the false ‘all’ (Kant) or ‘nothing’ (Locke, Berkeley, Hume) dichotomy on the innateness of 3D space, arguing for partial innateness instead. Third, it rejects cue integration accounts that see 3D vision as a trade-off between stereo vision and other depth cues (motion, perspective, and shading), arguing that such accounts fail to distinguish between perceived and depicted depth.

## Data Availability

This article has no additional data.

## Footnotes

1 Although the perceived depth need not be ‘geometrically’ correct: if you view figure 6*a* as a stereogram you seem to experience more depth than suggested by figure 6*b*.

2 One reviewer asks whether we need to control for attention as well. I don't think so. It's unclear how attention would change the spatial arrangement or shade of the letters on the page—and those seem to be the key aspects of our visual experience of the letters. But in any case, add that proviso if you want.

3 In a review of my book [41], Erkelens [73] suggests: ‘The division made by Linton between depth perception and depth judgments is intriguing, although I personally have the conviction that the division concerns different spaces. In my view, perceived depth relates to visual space and judged depth to physical space’. It's important to emphasize that under my account automatic cognition applies to both visual space (an inability to accurately introspect on our own visual experience, e.g. misjudging the size of the far car in figure 18*a* as 20% larger than the near car *in image size*) and physical space (far car as ×2 larger than the near car *in physical size*).

4 Indeed, even on orthodox accounts the scaling of disparity from vergence is thought to be imperfect ([98] finds a distance range of 53.5–214 cm is compressed to 60 cm–125 cm), so you would still experience accentuated depth at closer distances.

5 One reviewer suggested that the ground plane is often present in images. But is this really the case? Think about any TV series or film. How often do they really show an extended ground plane, and how often do we instantly gauge scale without it? Similarly, we've spent the last 2 years teleconferencing during a pandemic, where we only see the speaker's head and surroundings, but the absence of the ground plane has caused no ambiguities of visual scale.

6 This is the subject of [41], Ch.2.

7 This is the subject of [41], Ch.3.

8 In my book [41], I suggested vision might triangulate depth from sub-threshold blur (vs. the then emphasis on above-threshold blur [149,150]). The thought being, if accommodation can respond to sub-threshold blur, why not depth perception? Piloting this with stimuli in a Badal lens set-up has convinced me this is a mistake.
